# Mathematical Camera Array Optimization for Face 3D Modeling Application

**DOI:** 10.3390/s23249776

**Published:** 2023-12-12

**Authors:** Bashar Alsadik, Luuk Spreeuwers, Farzaneh Dadrass Javan, Nahuel Manterola

**Affiliations:** 1 Faculty of Geo-Information Science and Earth Observation (ITC), University of Twente, 7522 NB Enschede, The Netherlands; b.s.a.alsadik@utwente.nl; 2 Data Management and Biometrics (DMB), Faculty of Electrical Engineering, Mathematics and Computer Science (EEMCS), University of Twente, 7522 NB Enschede, The Netherlands; l.j.spreeuwers@utwente.nl (L.S.); na.manterola@gmail.com (N.M.)

**Keywords:** camera network, photogrammetry, optimization, constrained minimization, 3D model, face recognition

## Abstract

Camera network design is a challenging task for many applications in photogrammetry, biomedical engineering, robotics, and industrial metrology, among other fields. Many driving factors are found in the camera network design including the camera specifications, object of interest, and type of application. One of the interesting applications is 3D face modeling and recognition which involves recognizing an individual based on facial attributes derived from the constructed 3D model. Developers and researchers still face difficulty in reaching the required high level of accuracy and reliability needed for image-based 3D face models. This is caused among many factors by the hardware limitations and imperfection of the cameras and the lack of proficiency in designing the ideal camera-system configuration. Accordingly, for precise measurements, we still need engineering-based techniques to ascertain the specific level of deliverables quality. In this paper, an optimal geometric design methodology of the camera network is presented by investigating different multi-camera system configurations composed of four up to eight cameras. A mathematical nonlinear constrained optimization technique is applied to solve the problem and each camera system configuration is tested for a facial 3D model where a quality assessment is applied to conclude the best configuration. The optimal configuration is found to be a 7-camera array, comprising a pentagon shape enclosing two additional cameras, offering high accuracy. For those who prioritize point density, a 9-camera array with a pentagon and quadrilateral arrangement in the X-Z plane is a viable choice. However, a 5-camera array offers a balance between accuracy and the number of cameras.

## 1. Introduction

Face recognition is a biometrics recognition approach that involves recognizing an individual based on their facial characteristics or features. Interestingly, 3D face recognition is a system that takes advantage of the human face’s 3D geometric information. It uses data from 3D sensors to determine the shape of a person’s face and to validate his/her stated identity by matching geometric features extracted from the 3D reconstructed faces to recognize people against a dataset.

**Figure 1:**
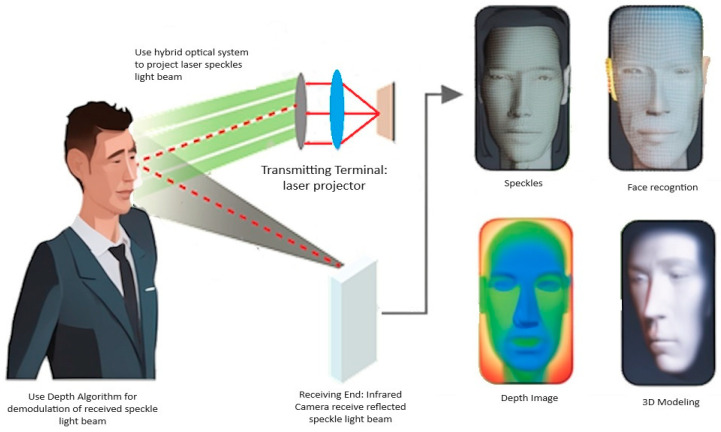
Face recognition using structured light technology [3].

By utilizing features that are not susceptible to lighting conditions, head orientation, varying facial expressions, and makeup, 3D face recognition has the potential to reach a higher accuracy than its 2D equivalent [1]. However, collecting the 3D face depth data can be achieved either using active sensors or passive sensors. Active sensing techniques can be based on laser triangulation [2], structured light [3], and time-of-flight [4,5]. The structured light technology (Figure 1) is based on using speckle images with particular coding to determine depth but some concerns such as sensitivity to ambient illumination and occlusions are currently being researched. By using numerous cameras, laser triangulation achieves submillimeter precision and prevents occlusions. However, it claims to capture periods of many seconds, like many other laser triangulation systems, rendering it inaccurate for scanning the face of a moving person. Time-of-flight systems currently have insufficient accuracy and information density to be used reliably for 3D face recognition [6].

On the other hand, passive sensors for 3D face recognition are currently thriving because of the advancements in using deep learning to enable a reconstruction from a single 2D image [7,8]. Still, the stereo vision using the camera array is the most reliable for 3D face modeling and recognition since it provides a realistic occlusion-free model. However, such a camera array system requires a sophisticated design to enable highly accurate 3D face modeling. Table 1 summarizes the advantages and disadvantages of the mentioned remote sensing techniques for 3D modeling.

Building a camera array system is necessary to ensure simultaneous capture of the face at the same instant to avoid any deformation in the reconstructed 3D model of such a nonrigid body. Therefore, further research on camera arrays designed for 3D face reconstruction is required to reach the high accuracy of active approaches as will be presented in this paper. Accordingly, the question that arises is how many cameras are enough for accurate facial 3D reconstruction? and in which reasonable configuration?

Currently, several multiarray camera systems are designed for the gaming and film industry, textile industry, medical industry, etc. like the examples in [22,23]. However, those systems require high costs, and large space, and may not fit facial modeling which requires a focused camera system. Using images for precise measurements and 3D modeling is a major task in the fields of computer vision, photogrammetry, and robotics.

In most of the mentioned image-based applications, it is required to have high geometric specifications including:

- Sufficient overlap percentage among an acceptable number of captured images.
- Suitable ray intersection geometry of the images defined by the base/height (B/H) ratio. The B/H ratio is an expression of the acceptable base distance B between the cameras themselves and the distance to the object H.
- Acceptable angles of incidence between the image rays and the object features.
- Pre-calibrated camera or pre-identified interior camera parameters.

Moreover, achieving optimal results necessitates favourable imaging conditions during image captures, including adequate scene illumination, stable capture free from shaking, and effective occlusion avoidance. These specified conditions collectively define the parameters of an optimal camera network.

The objective of having ideal or optimal camera networks is discussed several times in the literature [24,25,26,27]. The design task is aimed to be automatically applied to construct a robust network of overlapped images covering the required object and ensure reliability.

The method of the ‘next best view’ NBV represents the famous approach for the strategy of growing a few images into many [28,29]. The NBV method assumes a robot that only knows the position and the approximate dimensions of the object in question. Accordingly, the NBV search-based method is applied by adding one view (camera) selected among a set of candidate views and should fulfil some constraints related to visibility, accessibility, angle of incidence, and overlap. This NBV approach is iteratively applied by adding new views while the robot is navigating. However, the NBV methods pay more attention to the uncertainty at the robot positioning waypoints compared to the uncertainty at the object in the question itself.

Other research work was applied to find the ideal camera network based on the strategy of filtering many initial images to a minimum [30]. The filtering approach is based on an initial design of a very dense camera network around the rough point cloud of the object. This dens camera network is examined iteratively to indicate redundant images. In more detail, this filtering technique is based on the concept of having at least three images viewing the object points instantaneously. Hence, the redundant images are filtered out if they exclusively image only points that are covered by more than three cameras and then followed by an optimization step using the nonlinear-constrained minimization.

In this paper, we aim to find the best configuration of a camera array system for 3D face modeling using optimization techniques and recommend the most suitable one.

The paper sections are sequenced as follows: in Section 2, the proposed methodology will be explained in detail. In Section 3, 3D face modeling through optimized camera arrays will be presented. In Section 4, we will discuss the results and end up with the research conclusion.

## 2. Methodology

**Figure 2:**
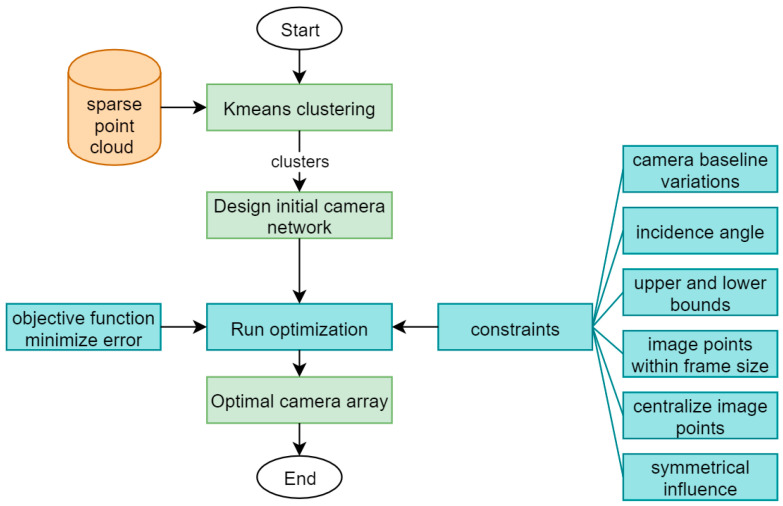
Proposed camera network optimization workflow.

As mentioned in the previous section, we propose a novel approach to a computerized camera network design that will conclude the optimal configuration of a camera array system for 3D face recognition. This will be applied by following the strategy of initializing a specific number of cameras and followed by mathematical optimization computations to fulfil the setup constraints against the required accuracy at the object space and stopping when required limits are reached. Therefore, a nonlinear constrained optimization starts from initial orientation values which are expected to converge rapidly to the global minimum solution. Figure 2 illustrates the proposed conceptual framework.

The developed optimization workflow as shown in Figure 2 will be designed to minimize the total error in the object points (Section 2.3.1). However, different constraints must be satisfied during the optimization as will be shown in detail in Section 2.3.2.

Mostly, the optimal camera network is constrained to different design requirements like the allowed B/H ratio which is highly contributing to getting an effective dense 3D reconstruction and accurate ray intersection. B/H is associated with the required ground sampling distance GSD, scale, camera angular field of view, and the required accuracy. It’s worth mentioning that the final aim of the imaging task will have a direct impact on the designed constraints of the optimization algorithm. For 3D face modeling, a short baseline network design is preferred where the B/H should be in the range of 15–30% [31,32]. On the other hand, wide baseline networks are designed for applications that require a high positional quality like structural deformation monitoring or laboratory camera calibration. Therefore, a B/H ratio of about 60% or greater is recommended. Another optimization constraint is the camera viewing angle or the angle of incidence which is of comparable importance to the B/H ratio as will be illustrated in Section 2.3.2.

### 2.1. Automated Initial Camera Network Design

**Figure 3:**
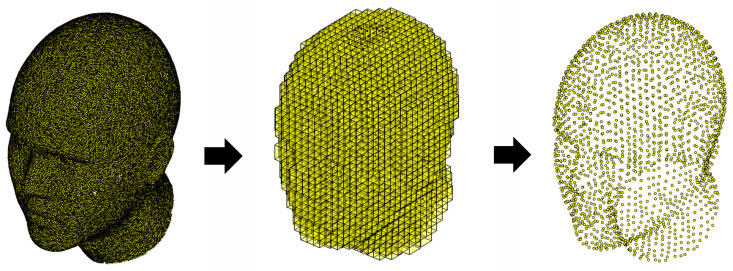
Point cloud downsampling. Left: Dense point cloud. Center: Voxels generated around points. Right: Downsampled point cloud obtained through voxel-based sampling.

To design an initial camera network, it is efficient to downsample the dense point cloud of the object. A uniform sampling approach is applied by the division of the dense point cloud into a regular grid of voxels. The size of voxels is determined by a specified sampling density or point spacing. All points falling within each voxel are considered as one group. The average of point positions (or center of mass) of the points within each voxel is computed. Then downsampled point cloud is formed by using these average points within each voxel (Figure 3). This uniform sampling method is intended to reduce the density of point clouds while preserving the structure of the face.

The rough point cloud of the object is then clustered into a specific number of clusters using k-means clustering where the points are partitioned in such a way that they are as close to one other as possible while being as far apart as possible from points in other clusters. This is done by minimizing the sum of distances between the cluster’s centroid and all of its points in each cluster. Accordingly, the total number of cameras required will be used to specify the number of point clusters.

Then for every cluster of points, the mean normal direction is calculated to define the optical axis of a viewing camera at an initial distance. Then the vector direction of the camera optical axis is converted into the rotation matrix M to complete the set of the six exterior orientation elements of each viewing camera.

To compute the rotation matrix M, first, we calculate the direction angle α of the initialized camera optical axis by using the dot product between two vectors $\overset{⃑}{a}$ and $\overset{⃑}{N}$ as in Equation (1):

(1) $$\cos\alpha =\vec{a}\cdot \vec{N}$$

The angles between the initial camera axis and the adopted XYZ coordinate system can be calculated using the cross-product between the mentioned normalized vectors as in Equation (2):

(2) $$\theta_{XYZ}=\overset{⃑}{a}\times \overset{⃑}{N}$$

**Figure 4:**
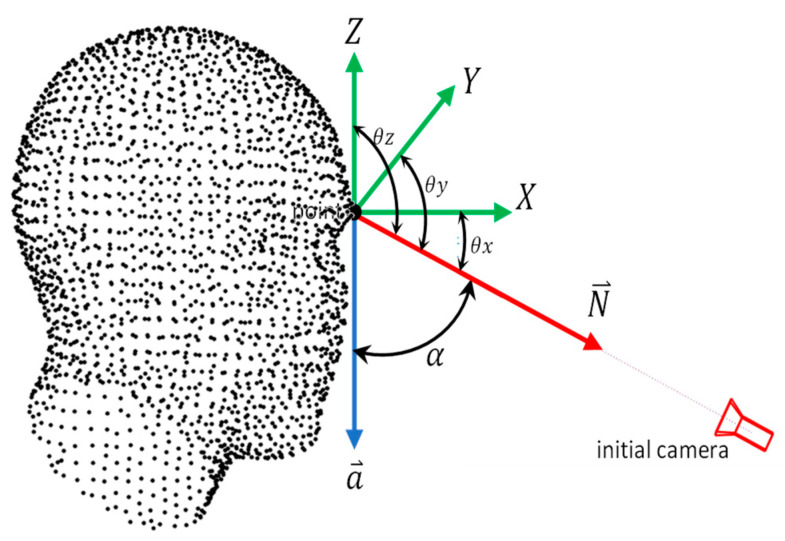
The initial camera rotation definition uses the normal pointing vector and Rodrigues rotation formula.

The described geometry is shown in Figure 4 where

$\overset{⃑}{a}$: The camera orientation in a nadir viewing $\left[\begin{array}{ccc} 0 & 0 & -1 \end{array}\right]$.

$\overset{⃑}{N}$: The normal direction vector of a cluster $\left[\begin{array}{ccc} n_{1} & n_{2} & n_{3} \end{array}\right]$.

$\theta_{XYZ}$: The angles enclosed between the normal vector and the three axes as $\left[\begin{array}{ccc} \theta x & \theta y & \theta z \end{array}\right]$.

Then, the rotation matrix defining the camera orientation in space can be derived using the following Equation (3) which is based on using Rodrigues’ Rotation formula [33,34].

(3) $$M=\left[\begin{array}{ccc} (1-cos\;\alpha ){\theta x}^{2}+cos\;\alpha & (1-cos\;\alpha )\theta x\;\theta y-sin\;\alpha \;\theta z & (1-cos\;\alpha )\theta x\;\theta z+sin\;\alpha \;\theta y\\ \left(1-cos\;\alpha \right)\theta x\;\theta y+sin\;\alpha \;\theta z & (1-cos\;\alpha ){\theta y}^{2}+cos\;\alpha & (1-cos\;\alpha )\theta y\;\theta z-sin\;\alpha \;\theta x\\ \left(1-cos\;\alpha \right)\theta x\;\theta z-sin\;\alpha \;\theta y & (1-cos\;\alpha )\theta y\;\theta z+sin\;\alpha \;\theta x & (1-cos\;\alpha ){\theta z}^{2}+cos\;\alpha \end{array}\right]$$

**Figure 5:**
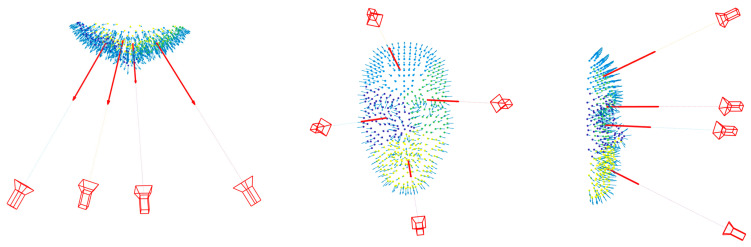
Illustration of initializing four cameras using the mean normal directions of four clusters.

Figure 5 illustrates a four-camera network initialization using four clusters of points.

### 2.2. Elements of the Mathematical Optimization

After the camera network initialization, optimization techniques will be followed. Optimization is generally formulated to compute a set of unknown parameters in a mathematical model $x=(x_{1},x_{2},\ldots x_{n})$ that can be defined as optimal. The optimization problem can be unconstrained in a simple case, this might be a minimization or a maximization problem. A more challenging optimization problem is found when the objective (cost) function f(x) to be minimized or maximized is subject to constraints in the form of equality constraints, $h_{i}\left(x\right)=0\;(i=1,\ldots .,m_{e})$, inequality constraints, $g_{i}\left(x\right)\leq 0\;(i=m_{e}+1,\ldots ,m)$; and lower $x_{l}$ to upper $x_{u}$ parameter bounds.

The solution of the nonlinear unconstrained minimization problem or nonlinear least-squares problem with redundant observations is either to be solved by Levenberg–Marquardt or by Gauss-Newton methods [35]. However, when the system of equations is constrained then it is harder to solve. Typically, the constrained minimization problem is solved by introducing the LaGrange multipliers λ composed of both quality $\lambda_{h}$ and inequality constraints $\lambda_{g}$ as follows in Equation (4):

(4) $$L\left(x,\lambda \right)=f\left(x\right)+\sum \lambda_{g,i}g_{i}(x)+\sum \lambda_{h,i}h_{i}(x)$$

The Karush-Kuhn-Tucker (KKT) conditions must be met to discover the optimal solution and ensure a global optimum for complicated minimization conditions [36].

It is worth mentioning that LaGrange multipliers λ convert the inequality constraints formulation into equality formulation in order to establish a stationary point where the partial derivatives are zero. As a result, in limited situations, generates a required condition for optimality.

Solving a large-scale nonlinear constrained minimization problem, as in the case of camera network optimization, is a difficult task. Trust region, sequential quadratic programming (SQP), and interior-point algorithms can be employed to address nonlinear-constrained optimization problems [36,37,38].

According to the literature, the interior-point technique has had a lot of success and has proven to be useful for a wide range of problem classes because of its regularization effects on the constraints. Because of their Newton-like properties in terms of scalability and convergence performance, interior-point methods have become the trusted solution method for large-scale optimization problems, according to [39,40]. As a result, the interior-point optimization technique will be used to tackle the camera network optimization problem in this research study.

### 2.3. The Formulation of the Camera Network Optimization Problem

The mathematical model that represents the core of the camera network design and relates the interior and exterior camera parameters to the object coordinates is the collinearity equations model as illustrated in Equation (5). It should be noted that the bundle adjustment method which is based on the collinearity equations is widely used when estimating the adjusted camera parameters and the object coordinates [41].

(5) $$\left.\begin{array}{c} F_{x_{A}}=-f\frac{m_{11}\left(X_{j}-Tx\right)+m_{12}\left(Y_{j}-Ty\right)+m_{13}\left(Z_{j}-Tz\right)}{m_{31}\left(X_{j}-Tx\right)+m_{32}\left(Y_{j}-Ty\right)+m_{33}\left(Z_{j}-Tz\right)}-x\\ F_{y_{A}}=-f\frac{m_{21}\left(X_{j}-Tx\right)+m_{22}\left(Y_{j}-Ty\right)+m_{23}\left(Z_{j}-Tz\right)}{m_{31}\left(X_{j}-Tx\right)+m_{32}\left(Y_{j}-Ty\right)+m_{33}\left(Z_{j}-Tz\right)}-y \end{array}\right]$$

where

$F_{x_{A}}$ and $F_{y_{A}}$ represent the differences between the observed image coordinates x and y and their computed values.

f: focal length.

x,y: image coordinates.

Tx,Ty,Tz: camera coordinates.

$X_{j},Y_{j},Z_{j}$: object point coordinates.

m’s: rotation matrix element derived from three angles $\left(\omega ,\varphi ,k\right)$ and based on a right-handed system.

As mentioned in the previous section, the most costly computational step is to solve the large-scale mathematical constrained minimization problem especially if the 3D face is represented by a large number of n points and with a very small tolerance for stopping criterion.

In summary, the optimization problem of the camera network design needs a precise definition of the input and output parameters which can be listed as follows:

**The input data parameters:**

Point coordinates defining the object ($X_{j},Y_{j},Z_{j},j=1:n$).

For every initial camera i, there are six initial exterior orientation parameters $x0=\left(\omega_{i}{}^{\circ},\varphi_{i}{}^{\circ},\kappa_{i}{}^{\circ},{Tx}_{i}{}^{\circ},{Ty}_{i}{}^{\circ},{Tz}_{i}{}^{\circ}\right)$. The parameters vector x0 represents the initial guess of unknowns for running the subsequent optimization step.

**The output parameters:**

The optimal exterior orientation parameters $\hat{x}={\hat{\omega }}_{i},{\hat{\varphi }}_{i},{\hat{k}}_{i},{\hat{Tx}}_{i},{\hat{Ty}}_{i},{\hat{Tz}}_{i}$ for each designed camera i in the whole camera array network.

It should be noted that between the mentioned input and output steps, there are many processing formulations regarding the cost function and the optimization constraints as will be discussed in the following sections.

#### 2.3.1. Cost Function

As mentioned, the objective of the optimization is to build a strong camera network that ensures minimum errors or higher accuracies at the object points. Accordingly, the cost function is formulated by computing the covariance matrix of the object points $Q^{s}$ using the least-squares adjustment method as shown in Equation (6) [21].

(6) $$Q^{s}={\left(B^{t}WB\right)}^{-1}=\left[\begin{array}{ccc} \sigma_{X}^{2} & & \\ & \sigma_{Y}^{2} & \\ & & \sigma_{Z}^{2} \end{array}\right]$$

where

B: the matrix of the partial derivatives of the collinearity equations concerning the object coordinates (X,Y,Z).

W: the weighting matrix.

$\sigma_{X}^{2}$,$\sigma_{Y}^{2}$,$\sigma_{Z}^{2}$: variances at *XYZ* coordinates respectively.

Accordingly, the cost function G is designed to minimize the norm of the eigenvalues $\left(\lambda_{1},\;\lambda_{2},\lambda_{3}\right)$ of the covariance matrix $Q^{s}$ as shown in Equation (7).

(7) $$G=\min\left(\left|eigen\;Q^{s}\right|\right)=min\left|\lambda_{1},\;\lambda_{2},\lambda_{3}\right|$$

where | | refers to the norm.

Since the eigenvalues represent the error ellipsoid axes lengths at each object point, this cost function of Equation (7) is meant to improve the accuracy of the whole camera network.

As mentioned, the camera optimization problem is nonlinear and needs to be constrained to obtain realistic results that satisfy the final goal of the imaging. In the next Section 2.3.2, an explanation is given about the necessary constraints involved in camera network optimization.

#### 2.3.2. Network Design Constraints

The camera network design problem is influenced by specific geometric constraints, which can be listed as follows:

The lower and upper bounds of the estimated parameters for each designed camera (Equation (8))

(8) $$\left.\begin{array}{c} \begin{array}{c} -90{}^{\circ}<\omega_{i}<90{}^{\circ}\\ -90{}^{\circ}<\varphi_{i}<90{}^{\circ}\\ -180{}^{\circ}<k_{i}<180{}^{\circ} \end{array}\\ \begin{array}{c} T_{xi}-Dx<T_{xi}<{T_{xi}+Dx}^{}\\ T_{yi}-Dy<T_{yi}<T_{yi}+Dy\\ T_{zi}-Dz<T_{zi}<T_{zi}+Dz \end{array} \end{array}\right\}$$

**Figure 6:**
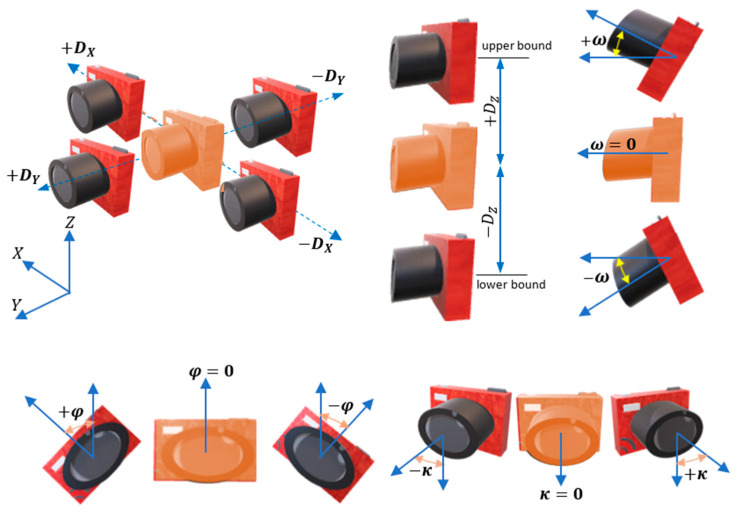
The bounding limits of the optimal camera orientation.

The allowed movement in the camera position Dx,Dy, and Dz depends on the design problem and the available space that can be occupied around or inside the object. Ground sample distance GSD is usually defined in the design requirements, and it has a direct relation with the scale and the camera bounds of Tx,Ty, and Tz. As shown in Equation (8), angles ω and φ have rotation bounds within ±90° while k bound is designed in the range ±180°. The bounding limits are illustrated clearly in Figure 6 where the initial camera is colored orange.

Nonequality constraint of the *B/H* ratio: The *B/H* ratio between the designed cameras and the object can be formulated as follows in Equation (9):

(9) $${{Min}_{B/H}<B/H<Max}_{B/H}$$

where

**Figure 7:**
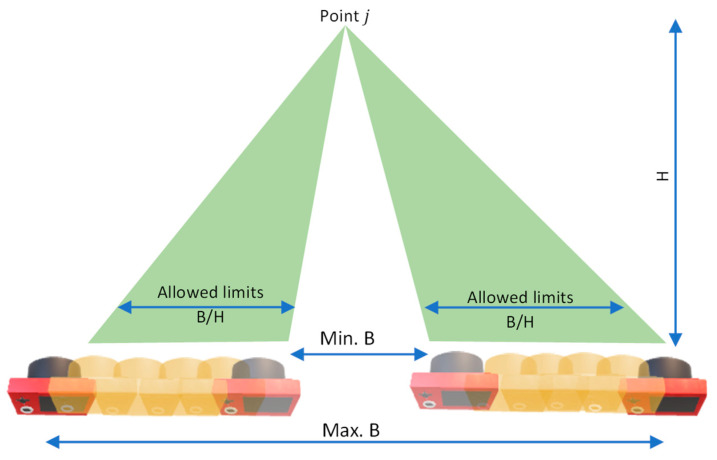
B/H constraint.

${{Min}_{B/H},Max}_{B/H}$: The minimum and maximum allowed *B/H* ratio (Figure 7).

$B=\sqrt{{{Dx}_{ik}}^{2}+D{y_{ik}}^{2}+{Dz_{ik}}^{2}}$ the base distance between camera i and k.

$H=\sqrt{{{∆x}_{ij}}^{2}+∆{y_{ij}}^{2}+{∆z_{ij}}^{2}}$ the distance between the camera i and the object point j.

A graphical illustration of the *B/H* constraint is shown in Figure 7 which shows the allowed *B/H* ratio within the upper and lower bounds while the camera orientation is changing during the optimization run.

**Figure 8:**
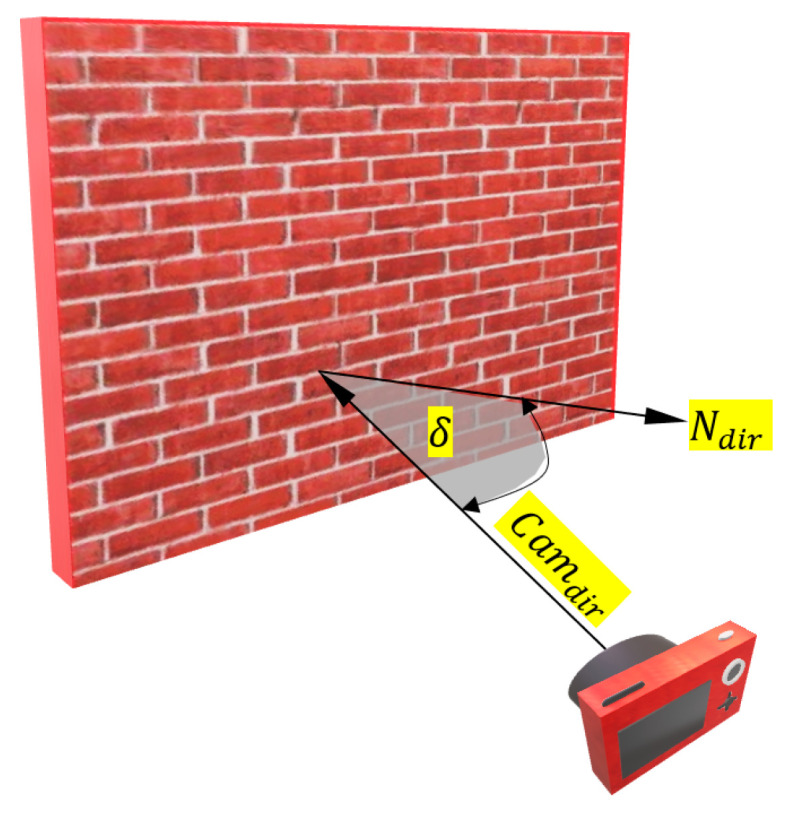
Incidence angle constraint illustration.

Nonequality constraint of the Incident angle: This constraint is formulated by computing the angle δ (Figure 8) between the object point normal and the designed camera optical axis as in Equation (10). The threshold angle can be 45° as an example regarding the network design’s final aim.

(10) $$\delta ={cos}^{-1}\frac{N_{dir}\cdot {Cam}_{dir}}{\left|N_{dir}||{Cam}_{dir}\right|}\leq threshold$$

where

$N_{dir}=$ normal direction of one object point.

${Cam}_{dir}=$ the camera axis direction

|| refers to vector length and ‘·’ refers to the dot product.

**Figure 9:**
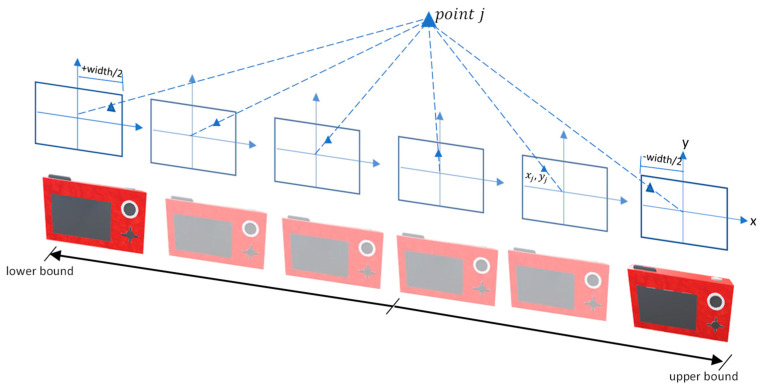
Illustration of the inequality constraint of the image coordinates in the x-direction.

Nonequality constraint of the image coordinates: Every image point j is constrained to remain observed from the same camera i during the optimization (Equation (11)).

(11) $$\left.\begin{array}{c} abs(x_{i})\leq width/2\;\\ abs(y_{i})\leq height/2 \end{array}\right\}$$

where height and width represent the image format height and width respectively (Figure 9).

Equality constraint of the image coordinate: This is intended to constrain the average of the image coordinates $\bar{xp}$, $\bar{yp}$ (in p.p. system) to equal zero (Equation (12)). This constraint is aimed at modifying the camera orientation to distribute the points evenly around the image center.

(12) $$\begin{array}{c} \bar{xp}=0\;\\ \bar{yp}=0 \end{array}$$

**Figure 10:**
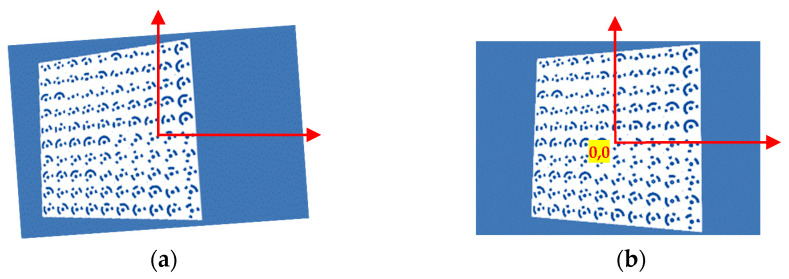
(**a**) without image equality constraints. (**b**) with image equality constraints.

The effect of this constraint is shown in the illustration of Figure 10 where the camera during the optimization can be rotated and/or translated to centralize the object points in the viewing image.

Equality constraint of the separation distances: This constraint is intended to maintain the predefined distance between the cameras. This is applied by running a Delaunay triangulation in the 3D space and constraining the length of the edges to the design separating distance (Equation (13)).

(13) $$mean\left(edge\_length\right)=design\;distance$$

Equality constraint of the symmetry pattern: This constraint is intended to have a semi-symmetrical pattern of the camera network. This is expected to comply with the manufacturing of a camera array system in a grid-like configuration especially knowing that the human face is almost symmetrical. The constraint is formulated to optimize the distribution of the cameras to ensure that the mean of their coordinates equals the median around the centroid of the object points (Equation (14)).

(14) $$\begin{array}{c} mean\;\left(T_{x}\right)-median\;\left(T_{x}\right)=0\\ mean\;\left(T_{y}\right)-median\;\left(T_{y}\right)=0\\ mean\;\left(T_{z}\right)-median\;\left(T_{z}\right)=0 \end{array}$$

Furthermore, the negative values of the camera coordinate are constrained to equal the positive values as shown in Equation (15).

(15) $$abs\sum {(T}_{x}<0)-abs\sum (T_{x}>0)=0$$

A final worthy to mention remark is that it can happen in the nonlinear-constrained minimization that the solution is well converged, and the step length is smaller than its threshold value while the constraints are not fully satisfied. We will consider this, if occurred, as an acceptable result since getting very close to the numbers we aimed for in the constraints meets our design requirements as well.

### 2.4. Pseudocode

To summarize the proposed minimal optimal camera network design workflow, a pseudo-code is given in Algorithms 1–3 (Pseudocode: summary of the proposed minimal optimal camera network design workflow) as follows:


**Algorithm 1:** Main program includes the input and output and call the optimization.functions of both: cost function and nonlinear constraints.Input:–  object points P(X,Y,Z)–  camera parameters: focal length, frame size, pixel size, lens distortion.–  initial camera orientation $\left(\omega {}^{\circ},\varphi {}^{\circ},k{}^{\circ},Tx{}^{\circ},Ty{}^{\circ},Tz{}^{\circ}\right)$ for 1:num. of camerascall Algorithm 2call Algorithm 3run nonlinear constrained minimzation using the interior-point method.Output: optimal camera orientation $\left(\hat{\omega },\hat{\varphi },\hat{k},\hat{Tx},\hat{Ty},\hat{Tz}\right)$Print results.



**Algorithm 2:** Compute the cost function of minimizing the *Q* matrix of the object points.Input: initial camera orientation and parameters, object points *P* and their normal directions.Output: cost function *F* min.eigen (*Q* covariance matrix)For *j* = 1:*P*For *i* = 1:no. of camerascompute rotation matrix *M*
compute image coordinates.endcheck visibility of *Pj* in camera *i*compute covariance matrix *Qj*endcost function *F* = |*eig*(*Q*)|



**Algorithm 3:** Compute the nonlinear constraints function of the camera design.Input: initial camera orientation and parameters, object points *P* and their normal directions.Output: nonlinear constraints [*c*,*ceq*]For *j* = 1:*P*For *i* = 1:no. of cameras     compute rotation matrix *M*.     compute angle of incidence *ij*.     compute image coordinates.     endcheck visibility of *Pj* in camera *i*endFor *h* = 1:no. of camerascompute *B*/*H* rationonequality constraints $c=\left[\left.\begin{array}{c} abs({xp}_{j})<width/2\;\\ abs({yp}_{j})<height/2 \end{array}\right\},{{Min}_{BD}<B/D<Max}_{BD}\right]$  equality constraints $ceq\;=\;\left[\begin{array}{c} \bar{xp}=0\;\\ \bar{yp}=0 \end{array}\right]$optional equality constraints *ceq* = *mean* (*T*) − *median* (*T*) = 0optional equality constraints *ceq* = *mean* (*edge_length*) = *design distance*nonequality constraints $\delta ={cos}^{-1}\frac{N_{dir\;}.{\;Cam}_{dir}}{\left|N_{dir}\right|\left|{Cam}_{dir}\right|}<threshold$end


### 2.5. Evaluation of the Optimization Algorithm

**Figure 11:**
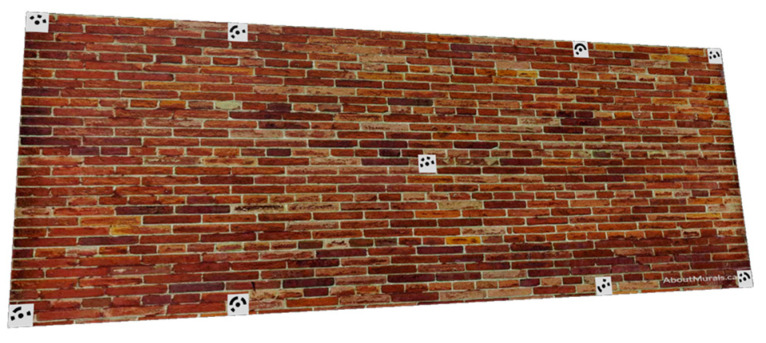
Nine coded targets are fixed on a wall.

To further illustrate the camera network optimization implementation, an example is given of a wall object (Figure 11) where nine well-distributed coded targets are placed on the wall where their coordinates are given in Table 2.

**Figure 12:**
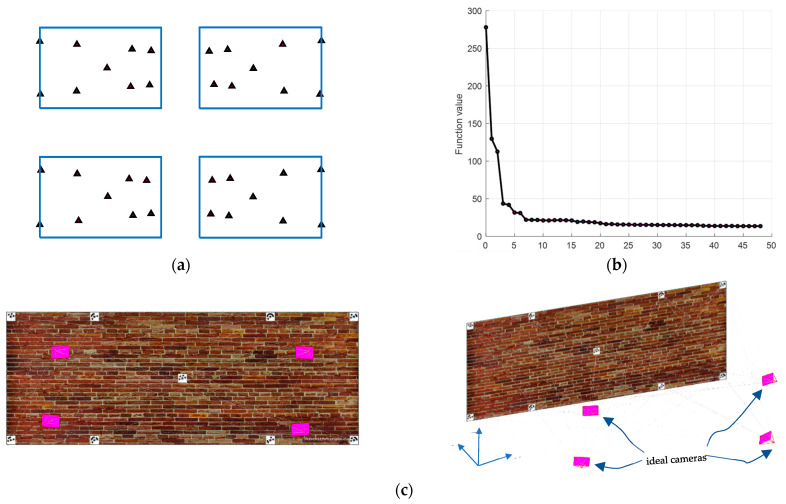

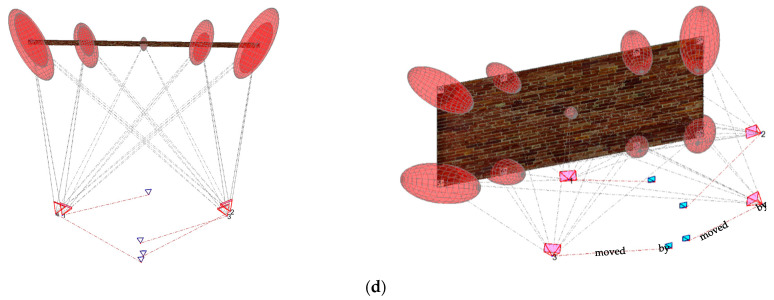
(**a**) the target image projections after optimization. (**b**) optimization solution run illustration. (**c**) optimal camera network. (**d**) exaggerated error ellipsoid at the target points after the initial cameras (cyan) oriented to their optimal orientation (magenta).

The example is designed to show the reader how a camera network consisting of four images will be optimized to ideal locations (Figure 12) that satisfy the following design constraints:

1- nonequality constraint of the image coordinates (Equation (11)).
2- equality constraint of the image coordinates (Equation (12)).
3- average B/H ≥ 0.6 and minimum B/H ≥ 0.2.
4- average incident angles ≤ 30°.
5- The lb and ub will be selected for angles in the range of ±45° from the initial values while in the range of ±15 m for Tx and Ty from the initial values.

The optimization started from a challenging initial camera orientation (Table 2 and Figure 12d) but robustly converged to the ideal configuration (Figure 12c) which meets the designed optimization constraints of minimizing the error ellipsoids at the coded target points (Figure 12d).

The image projections of the coded targets after optimization will appear as illustrated in Figure 11a while the optimization functional values are converged until all the constraints are satisfied and stopped when the step length becomes less than 1 × 10^−5^ (Figure 12b). The spatial distribution of targets across the entire image should reflect the favourable geometry of the camera array configuration, as illustrated in Figure 12a.

**Figure 13:**
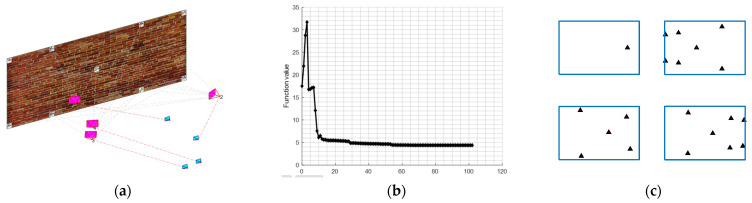
The optimization results when the inequality constraints of the image coordinates are neglected. (**a**) camera orientations before (cyan) and after optimization (magenta). (**b**) Cost function value iterations plot. (**c**) Targets image projections.

To illustrate further the impact of the constraints on the network design, we started to neglect some constraints in the optimization pipeline. First, the inequality constraint of the image coordinates of Equation (11) is neglected. Figure 13a shows the orientation result of the optimization where the cameras are wrongly moved closer to the wall and missed viewing most of the target points although the local minimum of the cost function is found (Figure 13b). The uneven spatial distribution of targets across the entire image coupled with one image missing displaying most of the target points highlights suboptimal results following optimization as depicted in Figure 13c.

**Figure 14:**
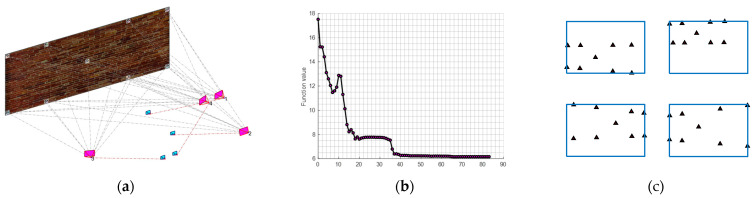
The optimization result when neglecting the equality constraints of the image coordinates. (**a**) camera orientations before (cyan) and after optimization (magenta). (**b**) Cost function value iterations plot. (**c**) Targets image projections.

When neglecting the equality constraints of the image coordinates (Equation (12)), the cameras are still oriented adequately (Figure 14a) and the optimization succeeded in converging to the global minimal where all the constraints are satisfied as shown in Figure 14b. However, when looking at Figure 14c we can notice how the target points are not any more well-distributed universally in the images as when the constraint is considered. Nevertheless, upon observing Figure 14c, it becomes evident that the target points no longer exhibit a uniform distribution pattern across the images.

**Figure 15:**
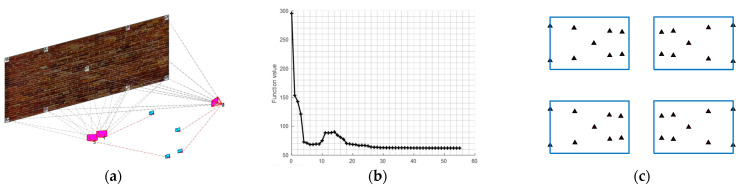
The optimization result when neglecting the minimum B/H ratio constraint. (**a**) camera orientations before (cyan) and after optimization (magenta). (**b**) Cost function value iterations plot. (**c**) Targets image projections.

Finally, when the minimum B/H ratio constraint is neglected, the optimization succeeds in converging to an optimal minimum, and the constraints are satisfied (Figure 15b). However, we can see how every pair of cameras are clustered close to each other (Figure 15a).

Accordingly, every suggested constraint mentioned in Section 2.3.2 is considered to be worthwhile in the camera network optimization algorithm.

## 3. Face 3D Modeling for Recognition

The following experiment is applied to assess the proposed optimal camera array design for 3D face modeling. A regular point cloud of a human head is used to design the proper camera network for a 3D image-based model.

To find the optimal configuration for a multi-camera system aimed at 3D face recognition, it has experimented to have four up to nine cameras mounted in a system that is supposed to capture instantaneously the images of an intended human face. All the mentioned optimization constraints will be implemented to minimize the error at the face object of interest.

**Figure 16:**
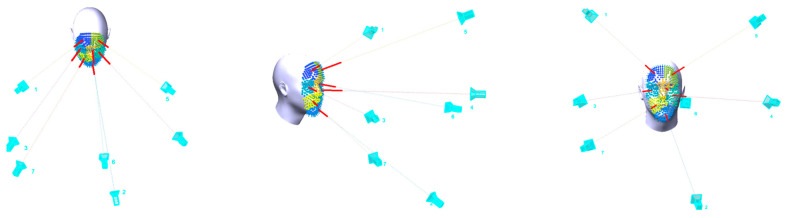
Initial camera orientation is based on using the normal directions (red lines) of the facial cluster of points.

**Figure 17:**
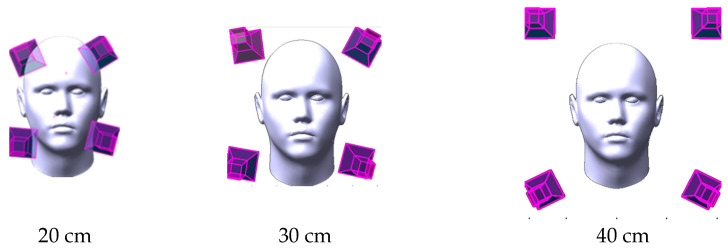
Camera array configurations are investigated using different baseline values to find the optimal configuration.

The face model of the human head of average dimensions 15 × 21 cm is freely shared in [42] as shown in Figure 16 and Figure 17. The experiment is applied on the head-derived point cloud and the camera array is designed for the optimization step.

The initial camera array has been shown (cyan) where the optical axis of each camera is initiated by the average normal direction (red lines) of every cluster of points. In Figure 15, seven cameras are initialized to view the facial cluster points. Those initial cameras will be reoriented using the nonlinear-constrained optimization algorithm described in Section 2.3.

In the following Figure 16, the optimal camera system configuration using a 20, 30, and 40 cm baseline respectively is shown. As mentioned, the optimal array configuration is computed for each number of cameras ranging from four up to nine cameras. Then after image capture, the 3D point cloud for each configuration is reconstructed. Worth noting that the imaging distance between the face and the camera array system will change according to the camera focal length setup.

The optimization algorithm will run to satisfy the objective function of minimizing the errors at the object points while satisfying the equality and nonequality constraints mentioned in Section 2.3.2.

**Figure 18:**
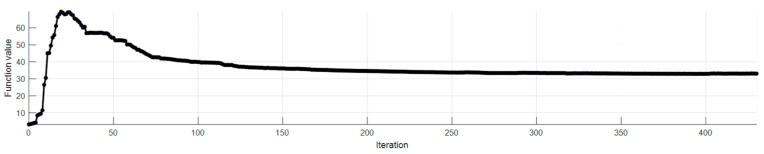
Optimization plot of the objective function for the 3D face reconstruction and recognition.

In Figure 18, the optimization graph is shown when applied using a camera array composed of 5 cameras and it stops when the size of the step is less than the value of the step size tolerance of 1 × 10^−5^.

**Figure 19:**
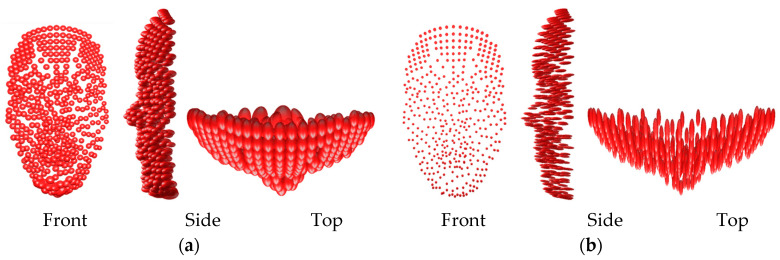
Error ellipsoids of the face exaggerated 10×: (**a**) before camera network optimization. (**b**) after camera network optimization.

The accuracy improvement expected after the optimization is visualized in Figure 19 by the ellipsoid of errors derived by adding a normally distributed noise of 1 cm to the image coordinates of the face points in the viewing cameras. As expected, the error ellipsoids are elongated in the depth direction (Figure 19a). The reason is the restricted small baseline of the camera array system (20–40 cm) compared to the wide baseline initiated from the clusters (Figure 16). On the other hand, the smaller baseline will ensure fewer occlusions and successful depth map reconstruction. Furthermore, in the initial camera design, some points may not be visible by at least two cameras while after optimization all the face points will be viewed by all the cameras.

**Figure 20:**
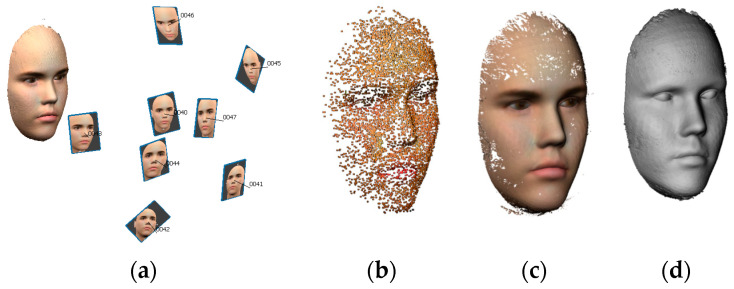
The image-based 3D modeling outputs using the Metashape tool [43]. (**a**) Automated image orientation. (**b**) Sparse point cloud. (**c**) Dense point cloud. (**d**) 3D mesh.

After finishing the optimization computations and the best configuration of the multicamera arrays is potentially found, experimentation is applied using a simulated environment in the blender tool. Figure 20 shows the image-based 3D modeling reconstruction steps.

**Figure 21:**
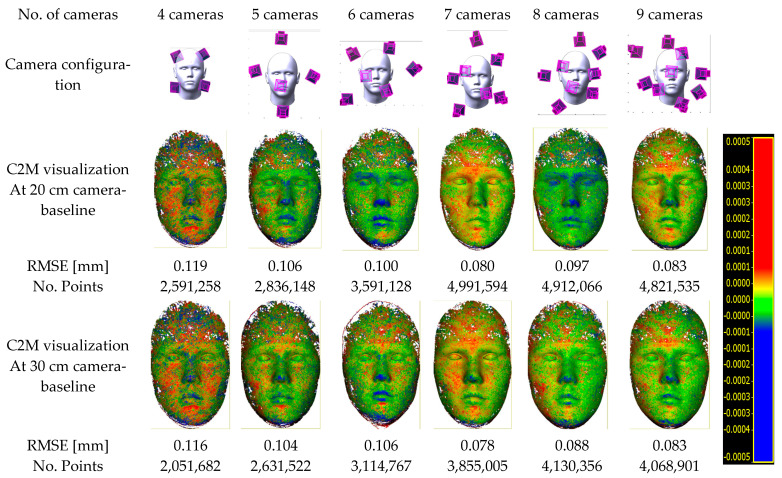
3D face reconstructed point clouds coloured according to the error distance (C2M) from the reference model out of the six types of optimal camera arrays in both cases of 20 cm and 30 cm baselines.

Worth mentioning that some referencing coded targets are placed close to the face to guarantee the correct orientation and scale concerning the ground truth model and to enable reliable comparison between all the produced 3D face models. In Figure 21, a summary of the experiment results is shown where two types of baselines of 20 and 30 cm are selected in the camera array design. The experiments started with four cameras and increased to nine cameras. The developed optimization algorithm can handle more cameras, however, we stopped at nine since we believe that having more cameras will increase the camera array size which we try to avoid and to have a compact system. In Figure 21, the distance between the created point cloud and the ground truth model is computed and visualized in a colour scale ranging from blue (−) to red (+). Furthermore, the number of points is also shown to indicate the sufficiency of the number of cameras when considered together with the distance measure.

**Figure 22:**
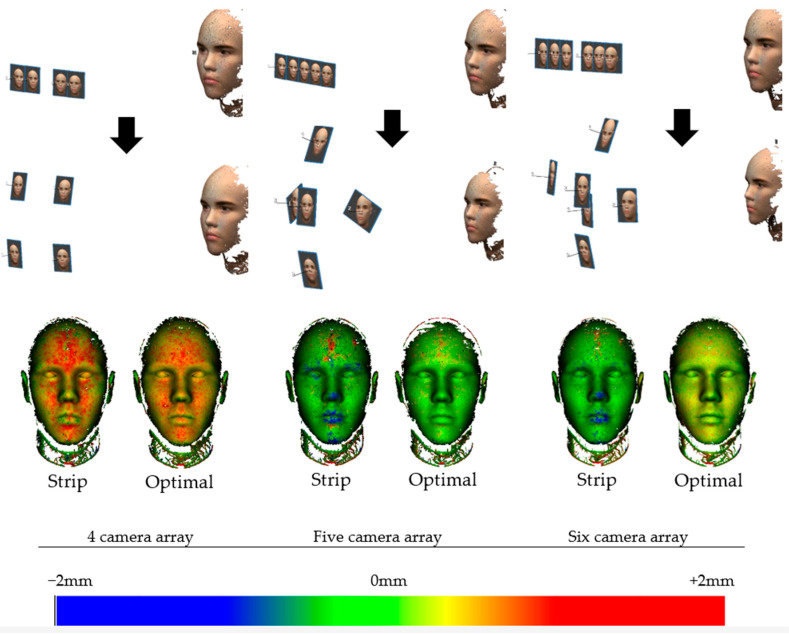
Comparison of 3D face point clouds obtained from a conventional image array capturing a strip and those generated by our optimal camera array algorithm. The colors indicate the error distance (C2M) from the reference model.

For validation, 4, 5, and 6 camera arrays are tested using conventional image array capturing a strip is utilized to generate a 3D face point cloud (Figure 22). Subsequently, this point cloud is compared to the one produced by our optimal camera array algorithm using an equivalent number of cameras. The reliability of the resulting 3D face models is assessed by comparing them against the ground truth model of our simulation as visualized in (Figure 22). This comparative analysis aims to validate the effectiveness of our optimal camera array configuration against the conventional imaging setup.

**Figure 23:**
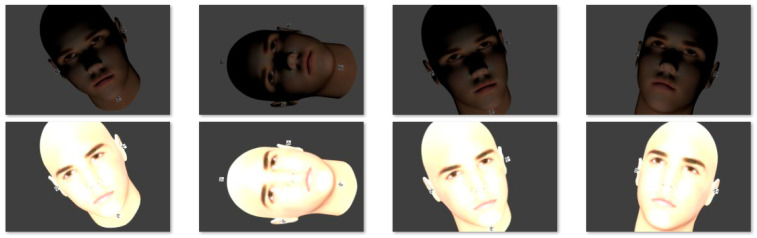
A sample of overexposed and underexposed sets of images.

**Figure 24:**
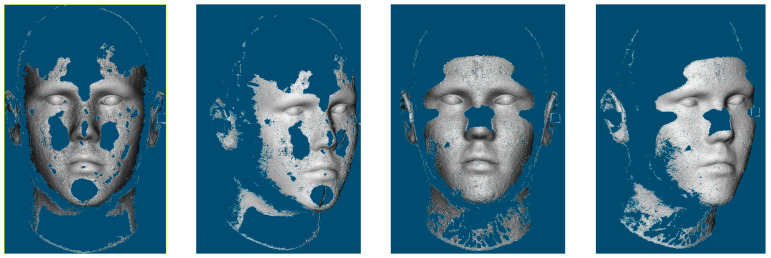
(**Left**) point cloud produced from the over-exposed images. (**Right**) point cloud produced from underexposed images.

Then, the produced point clouds are processed for the face recognition task according to the approach presented by Spreeuwers [44]. The applied 3D face recognition is highly successful in building the depth maps necessary for the recognition task in all the given camera configuration results. Accordingly, more challenging cases of using underexposed (dark) images and overexposed images are tested. An illustration is given in Figure 23 showing a sample of two sets of images. The reconstructed point clouds from the images in the two scenarios are shown in Figure 24.

The 3D face recognition approach is working successfully with a maximum score using the point clouds produced from overexposed images in all the camera configurations. However, the recognition approach fails with the point clouds produced from dark images due to the significance of missing parts.

Accordingly, the number of cameras does not affect the recognition results while illumination conditions do have a large impact on the reconstruction and recognition.

## 4. Discussion and Conclusions

In this paper, a novel approach is presented to find the optimal camera array suitable for 3D face recognition. The approach is based on a mathematical optimization technique where several design constraints are considered.

**Figure 25:**
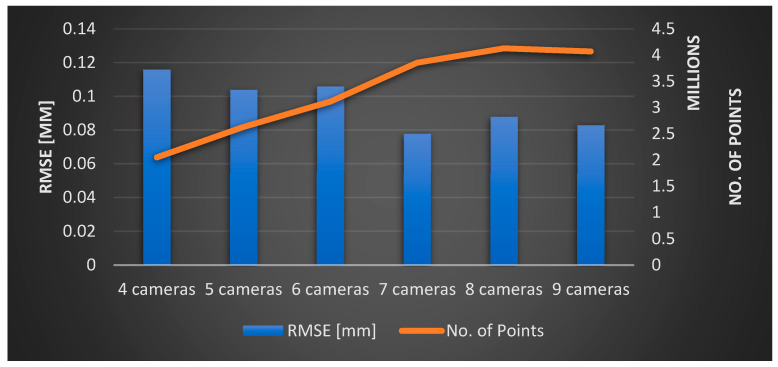
Performance comparison chart displaying accuracy and point density across various optimized camera array configurations.

Based on the optimization results, we can figure out what the camera array systems should look like using four cameras increasing up to nine cameras. Of course, increasing the number of cameras has advantages in terms of density and accuracy improvement and disadvantages in terms of being more susceptible to self-occlusions and expensive computations. As the number of cameras increases, the density of captured points and accuracy in the 3D face model tends to improve as illustrated in Figure 25.

The camera dimensions are selected to be compact similar to the GoPro camera of 6.2 × 4.5 × 3.2 cm and with a camera field of view of 26° at a 50 mm focal length to end up with a cost-effective system. As mentioned, the longer focal length will allow for a reasonable distance between the human face and the camera system while preserving the face to span the whole image frame. A summary of findings is listed below as:

When the camera baseline increased from 20 cm to 30 cm, the accuracy was slightly improved while the point density decreased. On average, a decrease of 10% to 30% in the point density was indicated while the accuracy remained generally at similar levels. If we consider having a more compact camera array system, then a 20 cm baseline is the preferred option.

In the optimization run, the stopping criteria are based on the selected tolerance threshold. Whenever the step size is smaller, the constraints will be better satisfied but a longer processing time is expected. However, if the constraint is satisfied to 0.01 mm using a threshold of 1 × 10^−5^ then it’s logical to prefer a threshold of 1 × 10^−6^ to satisfy the constraint to 0.001 mm since they both satisfy the required quality outcome.

Worth mentioning that all the shown designed camera arrays have reasonable dimensions of around 50 cm^2^.

**Figure 26:**
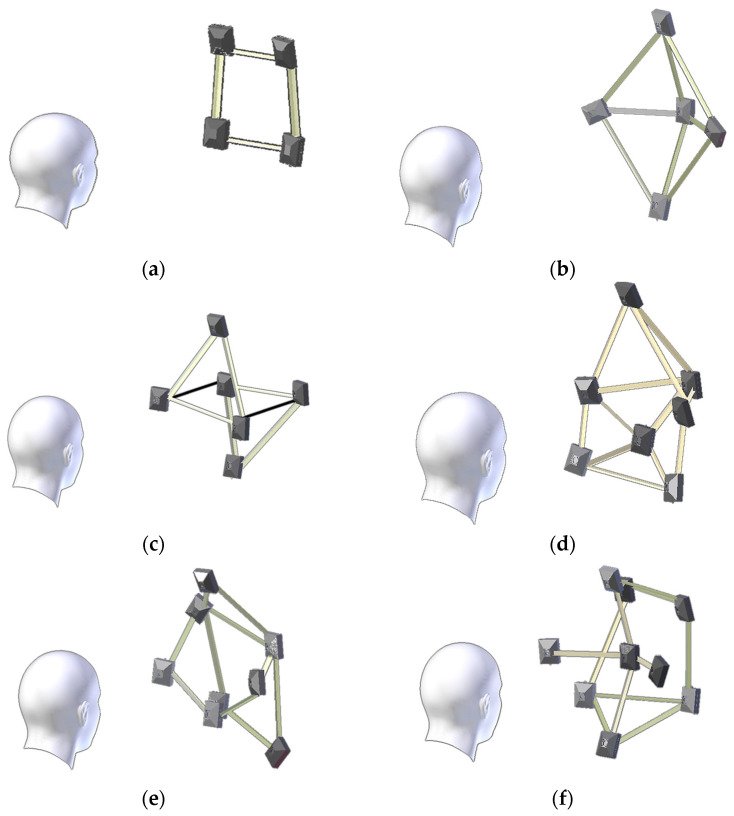
The concluded optimal camera array ranges from four to nine cameras for 3D face reconstruction and recognition. (**a**) optimal 4-camera array. (**b**) optimal 5-camera array. (**c**) optimal 6-camera array. (**d**) optimal 7-camera array. (**e**) optimal 8-camera array. (**f**) optimal 9-camera array.

The best constellation is found when using a 7-camera array which shows a high accuracy compared to the ground truth model in both baselines of 20 and 30 cm. This 7-camera array will be composed of a pentagon shape of five cameras enclosing the remaining two cameras (Figure 26d). More cameras will increase the density of points if preferred and then the best choice will be the 9-camera array which will be composed in the X-Z plane of a pentagon in front and a quadrilateral behind it (Figure 26f). Still, the 5-camera array is a good choice with a fewer number of cameras and high accuracy (Figure 26b).

It’s worth noting that illumination has a big impact on the success of face 3D reconstruction and recognition. Proper illumination ensures that facial features are well-illuminated and visible without shadows, highlights, or uneven lighting. This enables reliable and accurate reconstruction of the 3D face geometry. Accordingly, the proposed camera configurations will not be effective without having typical illumination conditions.

This research has important implications that will help 3D face recognition technology progress and be used in real-world applications. Our findings support cost-effective system design employing compact cameras, improve the accuracy and density of 3D points, and improve biometric verification and facial analysis performance. A framework is presented for camera system optimization in various applications as well as decision-making guidance for choosing appropriate camera array configurations. Several research drawbacks can be covered in future work, such as the lack of real-world experimentation, and the limited comparison with existing methods. Future work will investigate different conditions like varying illumination, skin tones, and facial expressions. Additionally, statistical analysis between the computed camera arrays and other conventional camera array approaches will be investigated.

Taking care of these issues will increase the practical application of our study’s findings.

## Figures and Tables

**Table 1:** Existing passive and active sensing techniques for 3D face modeling.

	Stereo Vision [9,10,11,12]	Structured Light [13,14,15]	Time-of-Flight (ToF) [4,16,17]	Depth Cameras [18,19,20,21]
**Advantages**	Provide accurate depth information from two or more cameras.	Provide high-resolution depth maps.	Direct measurement of light travel time allows for (Real-time).	Real-time depth information.
	Suitable for various environments.	Suitable for detailed 3D modeling	Performs well in low-light conditions.	Suitable for various applications.
	Preferred for high-precision applications.	Preferred for high-precision applications.		
**Disadvantages**	Requires good illumination conditions besides reliable geometric conditions.	Sensitive to ambient lighting	Limited accuracy at longer distances. Affected by ambient infrared light sources.	Limited accuracy Performance can be affected by environmental factors.

**Table 2:** The results of optimization example.

Initial							computed image coordinates [mm]									
omega [deg]	phi [deg]	kappa [deg]	X [m]	Y [m]	Z [m]			x-coordinates				y-coordinates				
90.12	5.51	0.00	1.00	−24.23	4.82		coded target	cam 1	cam 2	cam 3	cam4	cam 1	cam2	cam3	cam 4	
91.00	0.48	0.00	0.40	−35.74	5.80		point 1	−11.15	−9.42	−10.82	−8.49	4.79	3.01	4.80	3.05	
90.49	5.22	0.00	−0.82	−33.74	−0.85		point 2	9.51	11.15	8.27	10.90	3.08	4.94	3.00	4.89	
90.16	4.98	0.00	0.58	−36.45	1.15		point 3	8.72	10.85	9.45	11.15	−3.10	−4.81	−2.96	−5.00	
							point 4	−10.84	−8.43	−11.15	−9.33	−4.70	−3.04	−4.95	−3.02	
Given targes coordinates							point 5	1.18	−1.29	1.32	−1.32	−0.11	−0.13	0.15	0.11	
	X [m]	Y [m]	Z [m]				point 6	−4.25	−6.05	−4.33	−5.25	4.22	3.32	4.19	3.36	
point 1	−19.50	1.20	12.00				point 7	5.97	4.03	5.07	4.30	3.37	4.27	3.30	4.26	
point 2	19.50	1.20	12.00				point 8	5.29	4.33	6.13	4.04	−3.38	−4.21	−3.28	−4.31	
point 3	19.50	1.20	−2.00				point 9	−4.43	−5.17	−3.94	−5.99	−4.18	−3.34	−4.26	−3.35	
point 4	−19.50	1.20	−2.00				sum	0.00	0.00	0.00	0.00	0.00	0.00	0.00	0.00	
point 5	0.00	1.20	5.00				computed angular deviation [deg]									
point 6	−10.00	1.20	12.00					point 1	point 2	point 3	point 4	point 5	polnt 6	point 7	point 8	point 9
polnt 7	10.00	1.20	12.00				cam 1	21.78	21.78	21.78	21.78	21.78	21.78	21.78	21.78	21.78
point 8	10.00	1.20	−2.00				cam 2	24.58	24.58	24.58	24.58	24.58	24.58	24.58	24.58	24.58
point 9	−10.00	1.20	−2.00				cam 3	24.54	24.54	24.54	24.54	24.54	24.54	24.54	24.54	24.54
							cam 4	23.90	23.90	23.90	23.90	23.90	23.90	23.90	23.90	23.90
computed optimal orientataion							max(Ab) = 24 deg. < 30									
omega [deg]	phi [deg]	kappa [deg]	X [m]	Y [m]	Z [m]											
81.21	−21.09	0.00	−14.00	−28.64	5.82		results									
79.12	23.49	0.00	15.40	−27.69	7.93		B/H	constraint is min (B_H) > 0.2								
102.99	−24.20	0.00	−15.82	−27.25	−0.44		0.88	0.20	0.90	0.97	0.20	0.95				
99.15	23.85	0.00	15.58	−27.66	1.32		mean B_D = 0.69									

## Data Availability

Data is available upon request from the first author.
